# Enhancing neuroimaging genetics through meta-analysis for Tourette syndrome (ENIGMA-TS): A worldwide platform for collaboration

**DOI:** 10.3389/fpsyt.2022.958688

**Published:** 2022-08-18

**Authors:** Peristera Paschou, Yin Jin, Kirsten Müller-Vahl, Harald E. Möller, Renata Rizzo, Pieter J. Hoekstra, Veit Roessner, Nanette Mol Debes, Yulia Worbe, Andreas Hartmann, Pablo Mir, Danielle Cath, Irene Neuner, Heike Eichele, Chencheng Zhang, Katarzyna Lewandowska, Alexander Munchau, Julius Verrel, Richard Musil, Tim J. Silk, Colleen A. Hanlon, Emily D. Bihun, Valerie Brandt, Andrea Dietrich, Natalie Forde, Christos Ganos, Deanna J. Greene, Chunguang Chu, Michel J. Grothe, Tamara Hershey, Piotr Janik, Jonathan M. Koller, Juan Francisco Martin-Rodriguez, Karsten Müller, Stefano Palmucci, Adriana Prato, Shukti Ramkiran, Federica Saia, Natalia Szejko, Renzo Torrecuso, Zeynep Tumer, Anne Uhlmann, Tanja Veselinovic, Tomasz Wolańczyk, Jade-Jocelyne Zouki, Pritesh Jain, Apostolia Topaloudi, Mary Kaka, Zhiyu Yang, Petros Drineas, Sophia I. Thomopoulos, Tonya White, Dick J. Veltman, Lianne Schmaal, Dan J. Stein, Jan Buitelaar, Barbara Franke, Odile van den Heuvel, Neda Jahanshad, Paul M. Thompson, Kevin J. Black

**Affiliations:** ^1^Department of Biological Sciences, Purdue University, West Lafayette, IN, United States; ^2^Department of Psychiatry, Hannover University Medical School, Hannover, Germany; ^3^Max Planck Institute for Human Cognitive and Brain Sciences, Leipzig, Germany; ^4^Radiology Unit 1, Department of Medical Surgical Sciences and Advanced Technologies, University of Catania, Catania, Italy; ^5^University Medical Center Groningen, Department of Psychiatry, University of Groningen, Groningen, Netherlands; ^6^Department of Child and Adolescent Psychiatry, Technische Universität (TU) Dresden, Dresden, Germany; ^7^Department of Pediatrics, Herlev University Hospital, Herlev, Denmark; ^8^Department of Neurophysiology, Pitié-Salpêtrière Hospital, Sorbonne University, Paris, France; ^9^Pitié-Salpêtrière Hospital, Paris, France; ^10^Unidad de Trastornos del Movimiento, Servicio de Neurología y Neurofisiología Clínica, Instituto de Biomedicina de Sevilla (IBiS), Hospital Universitario Virgen del Rocío/CSIC/University of Seville, Seville, Spain; ^11^Centro de Investigación Biomédica en Red Sobre Enfermedades Neurodegenerativas (CIBERNED), Instituto de Salud Carlos III, Madrid, Spain; ^12^Department of Psychiatry, Psychotherapy and Psychosomatic, RWTH Aachen University, Aachen, Germany; ^13^Institute of Neuroscience and Medicine 4, Forschungszentrum Jülich GmbH, Jülich, Germany; ^14^JARA BRAIN—Translational Medicine, Aachen, Germany; ^15^Department of Biological and Medical Psychology, University of Bergen, Bergen, Norway; ^16^Shanghai Research Center for Brain Science and Brain-Inspired Intelligence, Shanghai, China; ^17^Department of Psychiatry, Medical University of Warsaw, Warsaw, Poland; ^18^Institute of Systems Motor Science, University of Lübeck, Lübeck, Germany; ^19^Department of Psychiatry and Psychotherapy, Ludwig Maximilians University of Munich, Munich, Germany; ^20^Deakin University, Geelong, VIC, Australia; ^21^Wake Forest School of Medicine, Winston-Salem, NC, United States; ^22^Department of Psychiatry, Washington University in St. Louis, St. Louis, MO, United States; ^23^Centre for Innovation in Mental Health, School of Psychology, University of Southampton, Southampton, United Kingdom; ^24^Radboud University Medical Centre, Donders Institute for Brain, Cognition and Behaviour, Nijmegen, Netherlands; ^25^Department of Neurology, Charité-University Medicine Berlin, Berlin, Germany; ^26^Department of Cognitive Science, University of California, San Diego, La Jolla, CA, United States; ^27^Department of Neurology, Medical University of Warsaw, Warsaw, Poland; ^28^Child and Adolescent Neurology and Psychiatric Section, Department of Clinical and Experimental Medicine, Catania University, Catania, Italy; ^29^Child Neuropsychiatry Unit, Department of Clinical and Experimental Medicine, School of Medicine, University of Catania, Catania, Italy; ^30^Department of Clinical Medicine, Faculty of Health and Medical Sciences, University of Copenhagen, Copenhagen, Denmark; ^31^Department of Clinical Genetics, Kennedy Center, Copenhagen University Hospital Rigshospitalet, Glostrup, Denmark; ^32^Department of Child Psychiatry, Medical University of Warsaw, Warsaw, Poland; ^33^Department of Computer Science, Purdue University, West Lafayette, IN, United States; ^34^Mark and Mary Stevens Neuroimaging and Informatics Institute, Keck School of Medicine, University of Southern California, Los Angeles, CA, United States; ^35^Department of Child and Adolescent Psychiatry/Psychology, Erasmus MC–Sophia Children’s Hospital, University Medical Center Rotterdam, Rotterdam, Netherlands; ^36^Department of Psychiatry, Amsterdam Neuroscience, Amsterdam UMC, Amsterdam, Netherlands; ^37^Centre for Youth Mental Health, University of Melbourne, Melbourne, VIC, Australia; ^38^South African Medical Research Council (SAMRC) Unit on Risk and Resilience in Mental Disorders, Department of Psychiatry and Neuroscience Institute, University of Cape Town, Cape Town, South Africa; ^39^Department Psychiatry, Department Anatomy and Neuroscience, Amsterdam University Medical Center (UMC), Vrije Universiteit Amsterdam, Amsterdam Neuroscience, Amsterdam, Netherlands

**Keywords:** Tourette syndrome, neuroimaging, genetics, ENIGMA, brain MRI

## Abstract

Tourette syndrome (TS) is characterized by multiple motor and vocal tics, and high-comorbidity rates with other neuropsychiatric disorders. Obsessive compulsive disorder (OCD), attention deficit hyperactivity disorder (ADHD), autism spectrum disorders (ASDs), major depressive disorder (MDD), and anxiety disorders (AXDs) are among the most prevalent TS comorbidities. To date, studies on TS brain structure and function have been limited in size with efforts mostly fragmented. This leads to low-statistical power, discordant results due to differences in approaches, and hinders the ability to stratify patients according to clinical parameters and investigate comorbidity patterns. Here, we present the scientific premise, perspectives, and key goals that have motivated the establishment of the Enhancing Neuroimaging Genetics through Meta-Analysis for TS (ENIGMA-TS) working group. The ENIGMA-TS working group is an international collaborative effort bringing together a large network of investigators who aim to understand brain structure and function in TS and dissect the underlying neurobiology that leads to observed comorbidity patterns and clinical heterogeneity. Previously collected TS neuroimaging data will be analyzed jointly and integrated with TS genomic data, as well as equivalently large and already existing studies of highly comorbid OCD, ADHD, ASD, MDD, and AXD. Our work highlights the power of collaborative efforts and transdiagnostic approaches, and points to the existence of different TS subtypes. ENIGMA-TS will offer large-scale, high-powered studies that will lead to important insights toward understanding brain structure and function and genetic effects in TS and related disorders, and the identification of biomarkers that could help inform improved clinical practice.

## Introduction

Tourette syndrome (TS) is characterized by multiple, persistent motor and vocal tics, and affects approximately 0.6–1% of children worldwide (1). Tics are often preceded by premonitory urges and resemble voluntary actions but are patterned and repetitive and may also be voluntarily suppressed. These characteristics suggest that neural networks and brain regions associated with both voluntary and involuntary motor behavior and affective processes may be involved. There is no cure for TS and efforts to develop novel pharmacological treatments are hampered by our limited understanding of the neurobiology and brain structural and functional deficits that underlie the disorder. With 90% of patients with TS presenting comorbid with other neuropsychiatric disorders, our efforts to understand and treat this disorder are further complicated. Most frequent comorbid disorders in TS are found along the impulsive–compulsive spectrum and include obsessive compulsive disorder (OCD up to 50% of patients with TS), attention deficit hyperactivity disorder (ADHD up to 54%), and autism spectrum disorders (ASDs up to 20%). Major depressive disorder (MDD up to 26%) and anxiety disorders (AXDs up to 36%) are also often found comorbid with TS, especially in the clinical settings (1–3).

From a genetic perspective, TS is a complex disorder; multiple genes interact with environmental factors to lead to the onset of symptoms (4, 5). Recent multi-site studies have identified genome-wide significant susceptibility variants and pathways that implicate ligand-gated ion channel signaling (highlighting the role of GABA), immune, cell adhesion, and transsynaptic signaling processes in TS (6–9). From a pathophysiology perspective, the quest for the anatomical structure and brain circuits that underlie TS and tackle its clinical heterogeneity has proven challenging, impeded by low sample size and efforts mostly fragmented across multiple sites (10, 11). Here, we present the scientific premise, perspectives, and key goals that have motivated the recent establishment of the ENIGMA-TS (Enhancing NeuroImaging Genetics through Meta-Analysis for TS) working group. Leveraging an international network of collaborators, existing collections of data, and established infrastructure and pipelines for large-scale neuroimaging and genetics studies, ENIGMA-TS will help close major gaps in understanding brain structure and function in TS, help dissect the basis of the heterogeneity of its clinical presentation, and explore the underlying links between TS and its frequently comorbid disorders.

### Leveraging the power of international collaboration to understand brain structure and function in Tourette syndrome

**FIGURE 1:**
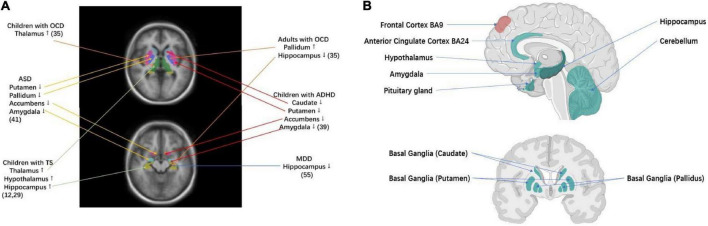
Brain regions that have been implicated in TS and related disorders **(A)** volumetric MRI studies of key brain regions for TS and related disorders. TS was associated with larger subcortical volumes of thalamus and hypothalamus (12, 29). OCD in children was associated with larger subcortical volumes of thalamus, while OCD in adults was associated with larger subcortical volumes of pallidum and smaller subcortical volumes of hippocampus (35). ADHD was associated with smaller subcortical volumes of the caudate, putamen, amygdala, and nucleus accumbens (39). ASD was associated with smaller subcortical volumes of the pallidum, putamen, amygdala, and nucleus accumbens (41). MDD was associated with smaller subcortical volumes of hippocampus (55). **(B)** TS–ADHD–ASD GWAS and TS–OCD GWAS cross-disorder tissue specificity analysis, testing 30/53 tissue types from GTEx v7 tissue expression atlas (61). Significant enrichment of gene expression in corresponding tissue under Bonferroni correction *(p* < 1.67 × 10^–3^ for 30 tissues tested and *p* < 9.43 × 10^–4^ for 53 tissues tested). The green label indicates enrichment of gene expression in TS–ADHD–ASD Tissue Specificity Analysis. The red label indicates enrichment of gene expression in both TS–OCD and TS–ADHD–ASD tissue specificity analysis (Created with BioRender.com).

To date, structural and functional neuroimaging studies for TS have been limited in size and have produced mixed results that are sometimes not replicated across studies [reviewed in (1, 5, 12–16)]. In general, abnormal development and/or maintenance of cortico-striato-thalamo-cortical (CSTC) circuits are implicated (1, 11, 17). Several studies report lower prefrontal cortical thickness in patients with TS (12, 18–22). Beyond the prefrontal cortex, structural alterations have been documented in many other brain areas, involving most brain structures associated with sensorimotor processing (13, 16, 23–29). The largest multicenter structural magnetic resonance imaging (MRI) study of TS to date (103 patients with TS and 103 matched controls), found lower white matter volume bilaterally in the orbital and medial prefrontal cortex, and larger gray matter volume in the posterior thalamus, hypothalamus, and midbrain in patients with TS (12) (Figure 1A). Similarly, studies of the functional neuroanatomy of tic disorders have been very limited in size. Resting-state functional MRI (rsfMRI) can be used to assess an idle “ticcing” state and inform on the brain networks associated with the manifestation of tics, irrespective of specific cognitive (task-related) demands (11). TS rsfMRI studies show reduced long-range connectivity and increased short-distance connectivity associated with motor processing (30, 31). Overall, these findings could reflect the aberrant or “immature” brain development of individuals with TS. However, these reports are based on a handful of patients and controls, and much larger studies are required to provide definitive evidence.

Small sample sizes have entailed low-statistical power and hampered the dissection of TS subtypes (on average less than 30 cases and 30 controls as reviewed in (11)). Furthermore, differences in analytic approaches have also contributed to inconsistent findings and limited reproducibility. ENIGMA (32) is an unprecedented worldwide initiative to tackle the crisis of reproducibility that comes from underpowered studies. Analyzing diverse worldwide samples (more than 40 countries and 1,400 investigators), ENIGMA has pioneered the identification of genetic markers that underlie cortical measures and subcortical volumes through neuroimaging genome-wide association studies (GWAS) (33, 34). Disorder-focused ENIGMA working groups have identified brain profiles for multiple psychiatric disorders and conditions based on an analysis of large worldwide datasets (32). These include the largest neuroimaging studies for multiple disorders that are highly correlated to TS: OCD (*N* = 5,423) (35–38) ADHD (*N* = 3,762) (39, 40), ASD (*N* = 3,222) (41), and MDD (*N* = 10,327) (42, 43) (Figure 1A).

The ENIGMA–TS working group was formed to address the need for large-scale studies to understand brain structure and function in TS and tackle clinical heterogeneity. The effort was motivated not only by ENIGMA but also TS–EUROTRAIN, an earlier collaborative consortium funded by the European Union (2012–2016), which represented an international network of researchers from 12 different sites in academia and industry aiming to understand the neurobiology of TS (5, 44–50). With a strong basis of collaboration, investigators from 23 sites and 12 countries (namely, Australia, Germany, Denmark, France, Italy, the Netherlands, Norway, Poland, Spain, the United Kingdom, China, and the United States) have already joined ENIGMA–TS and are currently working to pool and harmonize previously collected TS neuroimaging data with an initial goal of analysis for 1,000 TS individuals and a corresponding number of controls. The data mostly represent diverse European and European American ancestry and one cohort from China. There is an open and ongoing call for additional investigators to join ENIGMA–TS and information on how to join is available on the ENIGMA-TS website.^1^ Indeed, through our publications, conference presentations, our website, and access to the ENIGMA network, our goal is to draw additional membership, further expanding our sample size and representation of diverse populations in our studies.

The ENIGMA–TS working group uses standardized protocols for the processing and analysis of imaging data, to determine the reliability of effects across datasets, and identify common trends of low effect size that may not reach statistical significance in any individual study (37, 51, 52). We will use previously standardized methods to harmonize structural MRI, diffusion tensor imaging (DTI), and rsfMRI data across different sites, scanners, and imaging protocols (41, 53–55). In addition to greatly enhanced statistical power, data pooling allows novel comparisons across demographics (e.g., sex). In particular, our proposed analyses will have sufficiently large sample sizes to examine the effects of comorbidity and uncover different TS brain phenotypes or dimensions. Integrating with genetics, we will shed light on the links between TS and related disorders and underlying the brain structure and function (Figure 1B).

We can specify some hypotheses based on prior work, especially for structural MRI, which was the only case where sample sizes exceeded 100. The two largest previous structural studies in TS both found evidence for increased thalamic volume, especially in the posterior thalamus (12, 15). Several studies identified lower gray or white matter in the orbito-frontal cortex in TS (12, 18–20, 22, 56, 57). Others identified case–control differences in TS relating to the primary motor cortex (13). Studies of striatal volumes in TS have shown inconsistent results (12, 15), as have white matter DTI and functional connectivity rsfMRI results, but mostly included much smaller sample sizes compared to what we will achieve (13). However, rsfMRI has been demonstrated to contain diagnostically relevant information in TS (58). Several structural MRI reports suggest effects of age, sex, and comorbidity, supporting our plans to include such information in our models. In addition, both similarities and differences have been noted with the spectrum of childhood onset disorders that often occur comorbid with TS. For example, higher thalamic volume was also noted in the pediatric OCD (36, 59–62), whereas lower thalamic volume has been reported in ADHD (63–65). Lower gray or white matter volumes in the orbito-frontal cortex was seen in TS (9, 31, 58, 66–69) and also in the ADHD and OCD (18). Our hypotheses strongly motivate cross-disorder analyses, which we will also present later in this article.

### Genetics vs. brain structure and function in Tourette syndrome

**FIGURE 2:**
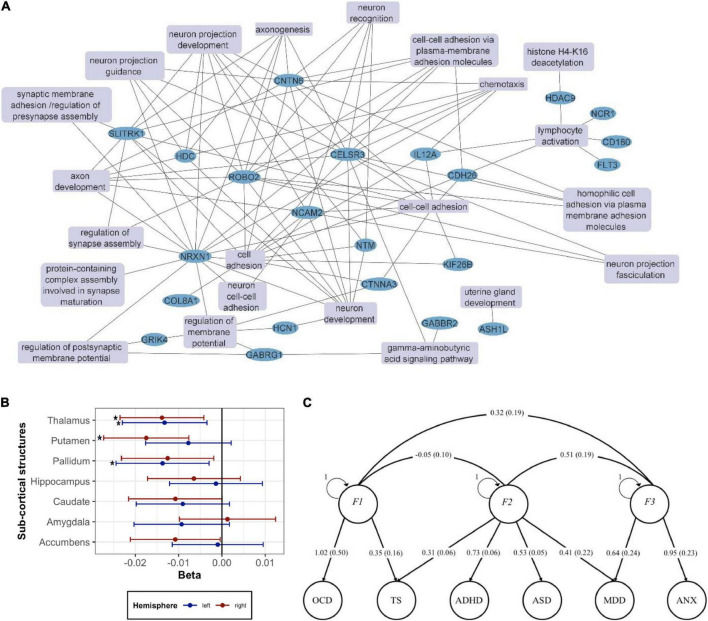
Genetics vs. brain structure in TS **(A)** network of the GO: Biological processes (GO:BP) terms from key genes previously implicated in TS as reviewed in the text. Enrichment analysis of the genes implicated in TS was performed with the ToppFun function in Toppgene (https://toppgene.cchmc.org/). Terms with *p* < 0.05 after FDR correction were considered statistically significant. Related GO:BP terms including the same genes were collapsed into a single term. Cytoscape (https://cytoscape.org/) was used for network visualization. The following genes were included: ASH1L (72), CD180 (7), CDH26 (7), CELSR3 (8, 70), CNTN6 (9), COL8A1 (71), CTNNA3 (93), FLT3 (7), GABBR2 (7), GABRG1 (7), GRIK4 (7), HCN1 (7), HDAC9 (7), HDC (74–76), IL12A (7), KIF26B (72), NCAM2 (7), NCR1 (7), NLRP7 (7), NRXN1 (9), NTM (7), ROBO2 (7), SLITRK1 (77, 78), WWC1 (8, 70). **(B)** Associations between TS PRS calculated based on the latest TS GWAS meta-analysis (79) and volume of 14 sub-cortical brain structures in UK Biobank. Each part was measured separately in the left hemisphere and the right hemisphere of the brain. Linear regression was performed with age, sex, genotyping batch, and top 10 PCs used as covariates in the analysis. The asterisk (*) indicates a significant association after multiple testing correction using the FDR method (*p* < 0.05). **(C)** Exploring the genetic architecture of TS and related comorbidities *via* genomic structural equation modeling. The path graph shows loads and corresponding standard errors in parenthesis.

The TS has a complex and heterogeneous genetic basis, with both common and rare variants contributing to risk (6–9, 70–78) (Figure 2A). The largest TS copy number variant (CNV) analysis performed to date (2,434 TS cases and 4,093 matched controls) (9), identified the first two genome-wide significant rare loci for TS (namely, *NRXN1* deletions, and *CNTN6* duplications) (9). The largest family exome-sequencing study for TS to date (800 trios), also pointed to *de novo* mutations that contribute to TS risk (8, 70), implicating two high-confidence TS risk genes, *WWC1* and *CELSR3*. On the contrary, focusing on common genetic variants, a large GWAS by Yu et al. (6) and follow-up studies showed that ligand-gated ion channel signaling, immune, cell adhesion, and transsynaptic-signaling processes are involved in TS (7). Analysis of an even larger GWAS for TS bringing together all major consortia working on TS genetics in a study of more than 12,000 patients is currently underway, promising novel insights into the genetics of TS by uncovering additional genes and pathways that underlie disease risk. Already, intermediate results point to two additional novel candidates for TS (79).

Bringing together TS GWAS and ENIGMA data, Mufford et al. performed the first study aiming to map TS genes to brain structure (80) using summary statistics from the Yu et al. TS GWAS (6) and the ENIGMA GWAS of subcortical volumes (30,717 individuals), we examined the genetic pleiotropy (the same SNP affecting two traits) and concordance (the agreement in SNP effect directions across these two traits). We found significant pleiotropy between TS and putaminal and caudate volumes, independent of the direction of effect, and significant concordance between TS and lower thalamic volume. It should be noted that this analysis associates TS with smaller thalamus, whereas the two largest previous studies in TS suggested an opposite effect. This discordance emphasizes the need for a much larger TS neuroimaging study, such as the one to be undertaken by ENIGMA–TS.

Furthermore, preparing for the ENIGMA–TS analysis, we asked whether the polygenic risk score (PRS) based on the recent Tsetsos et al. TS GWAS (79) correlates with brain structures in neuroimaging data from 29,798 individuals from the UK Biobank (UKB) (Figure 2B). We observed that increase in the genetic risk of TS was significantly associated with decrease in right putamen (*beta*: -0.0175, *adj.p*: 0.0069) and left pallidum (*beta*: -0.0137, *adj.p*: 0.043) volumes. We also found significant correlations between TS PRS and bilateral thalamic volume (*beta*: -0.0132 to -0.0138). ENIGMA–TS will pursue further analysis based on the most up-to-date TS GWAS and also the latest ENIGMA and UKB MRI GWAS datasets. In ENIGMA, more than 50,000 people from diverse populations from around the world have been assessed and analyzed with GWAS and whole-brain MRI, including over 10,000 with DTI and over 10,000 with rsfMRI. UKB data on approximately 10,000 individuals have also been recently integrated with ENIGMA (34, 81), while an even larger UKB dataset is now available. ENIGMA has now identified multiple genetic variants determining brain structure, namely, intracranial volume (ICV) (82) and subcortical volumes (33, 34, 83, 84). Our recent work also provides genetic determinants for regional and global measures of cortical surface area and thickness (34, 85). These rich datasets will be leveraged to gain insights into the brain structural measures that correlate with TS genetic risk.

### More than just tics: Understanding the genetic basis of frequent comorbidities in Tourette syndrome

With 90% of patients with TS presenting with additional neuropsychiatric comorbidities, understanding the molecular, pathophysiological, and neuroanatomical underpinnings of TS should also extend to investigating relationships to other comorbid disorders, with ADHD, ASD, OCD, MDD, and AXD being among the most prevalent (1–3). We recently performed the largest cross-disorder meta-analysis for TS, ADHD, ASD, and OCD, analyzing 124,000 samples and 6.8 million single nucleotide polymorphisms (SNPs) (66, 86). We showed that the hypothalamus-pituitary-adrenal (HPA) gland axis—and thus stress response—plays an important role in the shared pathophysiology of the studied disorders. Given the high comorbidity of TS to MDD and AXD, ENIGMA–TS will extend the cross-disorder analysis to include these additional comorbidities. Figure 2C shows our exploratory factor analysis of the genetic correlation matrix produced from multivariable LD Score Regression (LDSR), across the TS, ADHD, ASD, and OCD datasets described in Yang et al. (66) and large-scale GWAS on MDD and AXD (142,646 and 17,310 individuals, respectively) (87, 88). To do this, we used the R package, GenomicSEM (89). Identified factors highlight shared genetic liability across the studied disorders. We would like to point out the existence of a TS + OCD factor that is anticorrelated to ADHD and a shared liability factor with contributions across TS, ADHD, ASD, and MDD. Based on such analyses, we will pursue additional GWAS meta-analysis aiming to identify genetic susceptibility loci of different genetic factors along this phenotypic spectrum. We will then seek to correlate brain structure differences for the studied disorders to the genetic variants that underlie the brain structure in ENIGMA and UKB GWAS.

### Insights into Tourette syndrome pathophysiology from neuroimaging cross-disorder analysis

At a pathophysiological level, the association of tics with psychiatric comorbidities may result from the disruption of several cortico-basal ganglia loops. For instance, several behavioral and neuroimaging studies suggest the involvement of partly overlapping, albeit still separate, fronto-striatal circuits in both TS and ADHD (18, 22, 90). Although studies that compare TS to OCD have not been reported so far, the presence of OCD in patients with TS was found to be associated with volume reduction in the caudate nucleus (14), and lower cortical thickness in the ventromedial prefrontal cortex and hippocampus (22).

ENIGMA recently created pipelines that allowed a first cross-disorder analysis of cortical and subcortical brain structure across three of the disorders that appear often comorbid in TS (ADHD, ASD, and OCD) (54). Structural T1-weighted brain MRI scans of controls (*n* = 5,827) and individuals with ADHD (*n* = 2,271), OCD (*n* = 2,323), and ASD (*n* = 1,777) from 151 datasets worldwide were analyzed using standardized ENIGMA processing protocols. Subcortical volume and regional cortical thickness differences were examined in a mega-analytical framework (54). Analyses were performed separately for children, adolescents, and adults using linear mixed-effects models controlling for age, sex, and site (and ICV for subcortical measures). Lifespan dynamics were found in the pairwise findings: Children with ADHD compared with those with OCD had smaller hippocampal volumes, possibly influenced by IQ. Children and adolescents with ADHD had smaller ICV than controls and those with OCD or ASD. Adults with ASD showed thicker frontal cortices compared with controls and other clinical groups. No OCD-specific alterations across age groups—or surface area alterations among all disorders in childhood and adulthood—were observed. Furthermore, differences between medicated and unmedicated patients, and effects of duration of illness and age of onset were identified (54). Through collaboration with the relevant ENIGMA working groups, ENIGMA–TS will extend this work across TS, and ADHD, OCD, ASD, MDD, and AXD, analyzing a combined worldwide dataset of unprecedented power. Our work will yield brain maps of the main effects of each disorder, in children, adolescents, and adults, ranking brain metrics for effect sizes, and detection of metrics with common and disease-specific brain alterations.

### Validating Tourette syndrome genetic and neuroimaging biomarkers in population-based cohorts

Recent work supports a continuity of behavioral disorders–related traits across the population, with patients being at one extreme of the distribution. For instance, Demontis et al. found that ADHD symptoms in the general population are determined by largely the same genetic factors as those associated with a clinical diagnosis of ADHD (91). Robinson et al. showed that similar continuity from the general population to the clinical phenotype exists for ASD-related traits (92); also confirmed by others (93) and for OCD symptoms (94). In a similar manner, a recent study showed that PRS from the Yu et al. TS GWAS (6) predicted the presence of tics in a general population cohort (95). Such continuity thus allows us to extend the case–control findings to large population cohorts and to study the underlying mechanisms in more detail in terms of the roles of specific symptom domains and brain regions. More broadly, it also allows us to move toward the identification of diagnostic and prognostic biomarkers. ENIGMA–TS will seek to explore the value of the TS genetic and neuroimaging biomarkers that will be identified based on our studies to predict related symptoms in population-based cohorts. To do this, we will analyze large population studies (ABCD and Generation R cohorts) (96–100) for which symptoms and behavioral traits related to TS and its highly comorbid disorders of interest and genomic and MRI measures are available.

## Discussion

We have presented the background, rationale, and perspectives that support the establishment of the ENIGMA–TS working group and motivate our mission. Our large-scale, high-powered studies, integrating data from multiple countries, have the potential to offer a major breakthrough in the quest to understand brain structure and function and genetic effects in TS and correlated disorders. ENIGMA’s global approach offers higher power to detect factors that underlie TS onset and disease progression and test the generalizability of brain biomarkers in diverse samples across the globe. ENIGMA–TS already has partnerships with 12 countries while the ENIGMA reference neuroimaging GWAS includes samples from more than 40 countries around the world.

For the first time, ENIGMA–TS will undertake a large-scale cross-disorder study of brain structure and function and genetic susceptibility across TS and often comorbid OCD, ADHD, ASD, MDD, and AXD. We aim to identify biomarkers for disease subtypes that cut across diagnostic boundaries, lifting a major barrier in truly understanding factors that drive high comorbidity in patients with TS. Although our initial studies include mostly European datasets, we will make every effort to extend analyses to include representation from more diverse datasets through ENIGMA-available resources, public databases, and through our open call for additional collaborations.

To date, all major progress in understanding the genetics of TS and other neuropsychiatric disorders has been realized thanks to international collaborative efforts. Through ENIGMA–TS, we will seek to replicate this success to understand brain structure and function in TS and related disorders bringing together investigators working on the genetics and neuroimaging of these common disorders. Our joint work can offer far-reaching implications for future research, including the identification of robust multimodal markers of disease burden, and may ultimately lead to new therapies, improved patient management, and improved quality of life for patients and their families.

## Data Availability Statement

Publicly available datasets were analyzed in this study. This data can be found here: https://www.med.unc.edu/pgc/.

## Author contributions

PP designed the study and experiments, coordinated the study and data integration and analysis, and wrote the manuscript. YJ performed the experiments, analyzed and interpreted the data, and wrote the manuscript. KM-V, BF, OH, NJ, PT, and KB designed the study and experiments, contributed to the data, interpreted the results, and wrote the manuscript. All authors contributed data and methods, contributed to results interpretation, analyzed data, and wrote the manuscript.

## Footnotes

1
https://enigma.ini.usc.edu/ongoing/enigma-ts/
